# Dr. Sarah Howe Morris

**Published:** 1916-07

**Authors:** 


**Dr. Sarah Howe Morris** died at Santa Monica, Calif. in May, aged 84. It is said that she was the first woman to be graduated in medicine in the U. S. She was, for many years a practitioner of Buffalo, held in the highest esteem. She was an active member of the Homoeopathic County Society, the Women's Educational and Industrial Union which has recently turned over its property to the Arts Dept. of the University of Buffalo; and of the W. C. T. U. She removed from Buffalo about ten years ago.

